# What Is the Best Strategy for Enhancing the Effects of Topically Applied Ozonated Oils in Cutaneous Infections?

**DOI:** 10.1155/2013/702949

**Published:** 2013-10-27

**Authors:** I. Zanardi, S. Burgassi, E. Paccagnini, M. Gentile, V. Bocci, V. Travagli

**Affiliations:** ^1^Dipartimento di Biotecnologie, Chimica e Farmacia, Università degli Studi di Siena, 53100 Siena, Italy; ^2^Dipartimento di Medicina Molecolare e dello Sviluppo, Università degli Studi di Siena, 53100 Siena, Italy; ^3^Dipartimento di Scienze della Vita, Università degli Studi di Siena, 53100 Siena, Italy; ^4^Dipartimento di Fisiologia, Università degli Studi di Siena, 53100 Siena, Italy

## Abstract

Owing to diabetes, atherosclerosis, and ageing, there are several million patients undergoing skin lesions degenerated into infected ulcers with very little tendency to heal and implying a huge socioeconomical cost. Previous medical experience has shown that the daily application of ozonated oil eliminates the infection and promotes a rapid healing. The purpose of the study is the optimization of the antimicrobial effect of ozonated oils by testing *in vitro* four bacterial species and one yeast without or in the presence of different amounts of human serum. The results obtained suggest that a gentle and continuous removal of debris and exudate is an essential condition for the potent bactericidal effect of ozonated oils. In fact, even small amounts of human serum inactivate ozone derivatives and protect bacteria. The application of ozonated oil preparations is very promising in a variety of skin and mucosal infections. Moreover, ozonated oils are far less expensive than antibiotic preparations.

## 1. Introduction

There is a general convincement that ozone is one of the best compounds for killing bacteria, viruses, and parasites present in either dirty water or in prospectively useful drinking water [1–3], as well as against biofilms [4]. Although this is true, it has led to the assumption that intravenous injection of a gas mixture composed of oxygen (O_2_, ≥95%) and ozone (O_3_, ≤5%) in both bacterial sepsis and HIV patients will inactivate the pathogens and cure the diseases. Such a concept is wrong because it naively supposes that pathogens will be destroyed like those present in water, and in fact, the intravenous administration of O_2_-O_3_ has been prohibited because it is ineffective and prone to kill patients with O_2_ embolism [5]. On the other hand, during ozonated autohemotherapy (O_3_-AHT), ozone dissolves tenfold more than oxygen in the water of serum, but owing to the potent antioxidant capacity due to the presence of hydrophilic (uric acid, ascorbic acid, GSH, free Cysteine, and albumin) and lipophilic (vitamin E, bilirubin, thioredoxin, and *α*-lipoic acid) compounds, it is partly neutralized, while the bulk immediately reacts with n-3 and n-6 polyunsaturated fatty acids (PUFA) generating its crucial messengers: hydrogen peroxide (H_2_O_2_) and active aldehydes, mainly 4-hydroxy-2,3-trans-nonenal (4-HNE) [6]. Consequently, ozone having in blood an extremely short life cannot oxidize pathogens either free in exudates or intracellular because they are well protected by the serum and cellular antioxidants. Our previous paper [7] clarified that even the addition of only 5% human serum to the bacterial suspensions allowed bacterial survival in comparison to samples in saline tested with the same gaseous ozone concentration and time exposure. Such a result is important, and so far, it has been overlooked. 

Even at the risk of denaturing sensitive proteins, at least a partial disinfection of human serum can be performed *in vitro* with very high ozone concentrations. However, such conditions are not usable on the whole blood because of inherent blood cell damage. At the therapeutic range of both useful and safe ozone concentration for performing O_3_-AHT, the maximal H_2_O_2_ concentration can be about 40 *μ*M, but it cannot display bactericidal activity because it has a half time less than 1 min and a very fast dilution into the intracellular water of blood cells. These data justify the very modest activity of O_3_-AHT in bacterial and viral septic patients, not due to a direct anti-infective effects, but to a slightly enhanced immune activity elicited by the production of interferon and other cytokines induced by H_2_O_2_ in lymphomonocytes [8].

On the other hand, the direct ozonation of vegetable oils with unsaturated fatty acids leads to the formation of the 1,2,4-trioxolane moiety [9, 10], which represents the active form of ozone in these substrates. The trioxolane ring within the vegetable ozonated matrices quickly generates some compounds responsible for the healing process when applied in either a humid wound or an ulcer [11–14]. Moreover, it is accountable for antimicrobial and antimycotic treatments [15–17]. All these effects occur in the absence of cutaneous adverse reactions.

The main object of the present paper has been to clarify the antibacterial effectiveness of ozonated oils in mucosal and cutaneous infected wounds and ulcers which interest millions of patients who experience great discomfort and a relevant social-economic cost. Nonetheless, even in such a case, there is caveat because wounds and ulcers are always accompanied with an infection implying the presence of exudates comprising serum proteins, hence antioxidants, which may limit the efficacy of the ozonated oil. Sesame oil was selected for its wide use in pharmaceuticals as well as for its chemical compositions in terms of unsaturated fatty acids, with a balance between oleic and linoleic acid [12].

## 2. Materials and Methods

### 2.1. Materials

Chemicals were purchased from Sigma-Aldrich and used without further purification. In particular, the sesame oil (SO) was obtained from the seeds of *Sesamum indicum* (batch number S3547).

### 2.2. Ozonated Oil

SO was treated as reported in Sega et al. [10] in order to obtain the ozonated sesame oil (OSO) samples. Briefly, O_3_/O_2_ mixture was bubbled for different times in Drechsel bottles containing 40 mL of sesame oil, leading to different O_3_ amounts. The O_3_ flow-rate was kept constant at 1.5 L/minutes in all the experiments, and O_3_ concentration as evaluated in the feed gas was 45 mg/L. Chemical characterizations (namely, PV, peroxide value; AV, acidity value; IV, iodine value) of OSO samples have been performed. As for PV evaluation, it was determined by means of iodometric titration placing the sample at reflux for 60 minutes [18]. According to the PV, OSO has been classified as low (l-OSO), medium (m-OSO), and high (h-OSO). Viscosity measurements (Viscomate VM-10AL, CBC Europe) have been also performed by at both the temperatures of 22 and 35 ± 0.2°C. In Table 1, the physical-chemical characterization of the various test compounds is specified.

### 2.3. Microorganism Strains, Sample Preparations, and Culture Conditions

The reference strains of *Staphylococcus aureus* (ATCC25923), *Enterococcus faecalis *(clinical isolate), *Pseudomonas aeruginosa* (ATCC27853), *Escherichia coli* (ATCC25922), and *Candida albicans* (ATCC90028) used for this study were purchased from Oxoid.

On the basis of preliminary experiments, the evaluation of the antibacterial activity of OSO has been done either at 10^7^ CFU mL^−1^ or 10^4^ CFU mL^−1^ bacterial concentrations.

For the first line of experiments (microorganisms in contact with OSO at different content of peroxides), microorganisms from an overnight culture in tryptic soy agar (Oxoid) were suspended (density of 0.5 McFarland standard) in buffered physiological solution pH 7.4 (denominated saline) with Tween 80 (2%) and diluted in order to obtain about 10^7^ CFU mL^−1^. The addition of Tween 80 is indispensable for achieving a stable emulsion of oil in saline, and it is compatible with the microbial growth [19]. The samples were subdivided (5 mL) and introduced in centrifuge tubes containing different amounts (25 or 50 mg) of the oils (l-OSO; m-OSO; h-OSO) under investigation. 

For the second line of experiments (microorganisms in contact with h-OSO in the presence of different serum concentrations), microorganisms from an overnight culture in tryptic soy agar (Oxoid) were suspended (density of 0.5 McFarland standard) in buffered physiological solution pH 7.4 (denominated saline) with Tween 80 (2%) and diluted in order to obtain about 10^4^ CFU mL^−1^ in the presence of different serum concentrations (0; 5%; 10%). The samples were subdivided (5 mL) and introduced in centrifuge tubes containing 100 mg of sample oil, h-OSO.

In both experiments, the centrifuge tubes were shaken for 6 hours. For each treatment, 100 *μ*L was removed at different time intervals (1, 3, and 6 hours) from the tube and incubated for 24–48 h at 36°C. For each exposure time, the average number of colonies from treated plates was divided by the number of colonies from control plates to obtain a percentage viability value. Each treatment was repeated at least five times (CV% < 5).

### 2.4. SEM Characterization

The morphology of microorganisms before and after oil treatment (first line experiment, after six hours) was investigated by Scanning Electron Microscopy (SEM) studies. A drop of liquid cell suspension was placed on poly-l-lysine treated glass coverslip for five minutes. Then, the coverslip was fixed for immersion in a 2.5% glutaraldehyde solution in phosphate buffer 0.1 M pH 7.2 (PB) for 2 hours at 4°C, washed in PB, postfixed in 1% OsO4 in PB for 30 min. at 4°C, dehydrated in ascending alcohol series, incubated for three times in tert-butanol, and finally freeze dried. Afterwards, the coverslip was mounted on aluminum stub, coated with 20 nm gold in Balzers MED 010 sputtering device, and observed in Philips XL20 scanning electron microscope at 20 kV.

### 2.5. Statistical Analysis

Results were obtained from at least five independent measurements and expressed as the mean ± SD, unless otherwise stated. Statistical evaluations were performed by a one-way analysis of variance (ANOVA) using a statistics software (InStat software, version 3.0, GraphPAD Software Inc., San Diego, CA). Bonferroni test was employed after ANOVA to evaluate statistical difference between individual means. Significance was defined as a *P* value of less than 0.05.

## 3. Results

Table 2 shows the bactericidal effect with respect to time (1, 3, and 6 h) of different amounts (25 mg and 50 mg) of l-OSO, m-OSO, and h-OSO dispersed in the bacterial suspensions at about 10^7^ CFU mL^−1^. As it was expected, it has been possible to observe a concentration-dependent disinfectant trend. However, differences in behavior between the various strains tested at the different experimental conditions have been detected. In detail, no viable bacteria were obtained only after six hours and at the maximum peroxide content of the ozonated oils, except in the case of the less amount of OSO for *E. faecalis* that appeared to be the most resistant strain. Considering all the data, also *P. aeruginosa* and, to a lesser extent, *C. albicans* were quite resistant. On the contrary, *S. aureus* appeared to be the most sensitive one, with a sensible growth diminution since after the first hour with the minimum content of both ozonated oil and peroxide content. As regards *E. coli*, after three hours, a marked sensitivity to treatment has been observed, regardless of the amount of the ozonated oil. 

**Figure 1:**
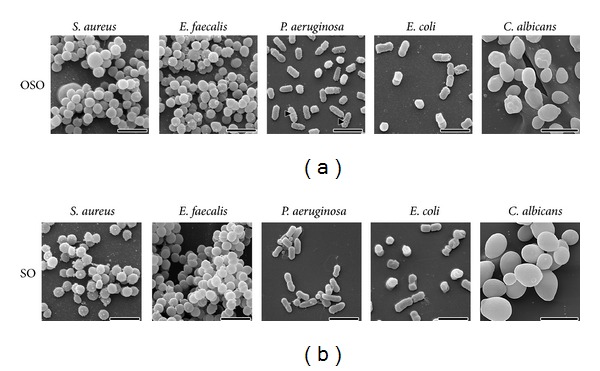
Scanning electron micrographs of the surface morphology of the cells after contact with either ozonated sesame oil (a) or sesame oil as control (b). Scale bars correspond to 2 *μ*m, except for *Candida albicans* (5 *μ*m). Arrowheads show small vesicles on cellular surface of *Pseudomonas aeruginosa* (see text for further details).

In order to have more information on the mechanism of degradation, S.E.M. investigation has been performed [20], and the results are shown in Figure 1. As it is possible to observe, both bacterial and *C. albicans* cells maintained intact shapes and size, just after ozonated oil exposition. Also the surface morphology of the cells was unaltered with respect to untreated ones, as well as the number of damaged cells. The only exception occurred at cellular surface of *P. aeruginosa* where the cells showed a rough outside with the appearance of tiny bumps similar to small vesicles, after contact with ozonated oil (Figure 1, arrowheads). 

Moreover, to simulate the *in vivo* conditions of application of the ozonated oils and to evaluate both if and how much the presence of cutaneous infection exudates can compromise the ozonated oil efficacy, the bactericidal effect with respect to time (1, 3, and 6 h) of 100 mg of h-OSO dispersed in the bacterial suspensions at about 10^4^ CFU mL^−1^ either in the absence or in the presence of serum at different concentrations (2.5%, 5%, and 10%) has been studied. For completeness' sake, blood and plasma are unsuitable to be tested because in the presence of bacterial suspensions they tend to coagulate. On the contrary, human serum while having a comparable amount of antioxidants does not present these drawbacks. 

As previously stated, the *in vitro* use of SO and its derivatives needs the emulsification with a surfactant, like the nonionic one Tween 80. Such experimental conditions have been calibrated after preliminary tests in order to obtain the best antibacterial effect against *E. faecalis* in the presence of about 10^4^ cfu/mL, assuming that such a quantity corresponds to 10^7^ cfu/g of infected cutaneous lesions [21].

**Figure 2:**
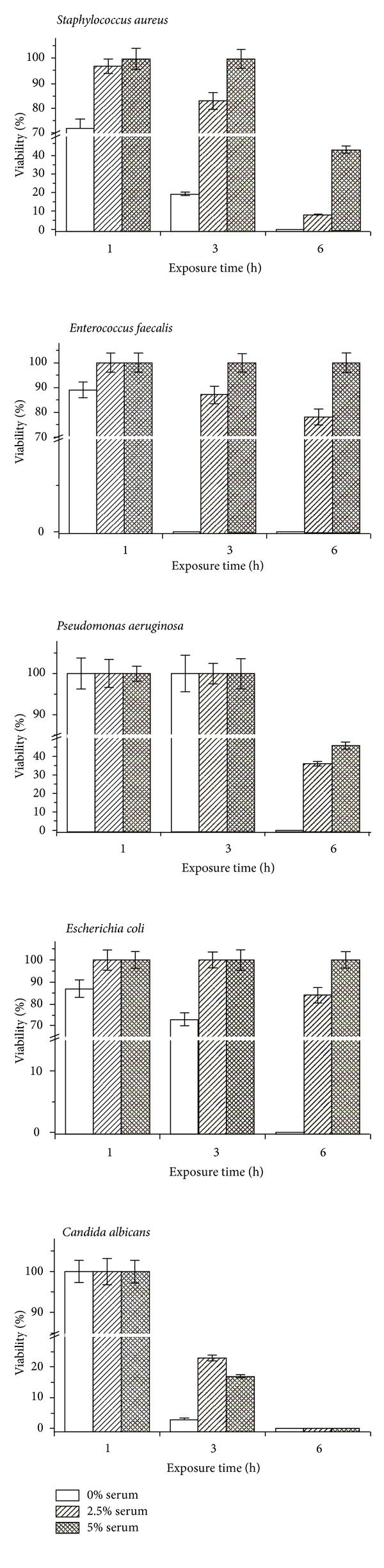
Viability of the treated cells with respect to the control after different exposure times to ozonated oil at the highest peroxide value either in the absence or in the presence of serum at different concentrations (see text for further details).

Figure 2 shows that the presence of human serum as low as 2.5% increased microorganism survival even at higher concentration of the oil at the higher peroxide content, indicating the role of protective biomolecules as antioxidants present in serum. Moreover, the bacterial viability totally remained when samples have been added with 10% of fresh serum (data not shown). However, in patients after the appropriate elimination of exudates, the ozonated SO is charged every 12 hours, and its therapeutic activity is likely to be more effective. We are planning to evaluate the effect of such ozone derivatives *in vivo* in a clinical trial.

## 4. Discussion

It is known that ulcers with scarce tendency to heal are due to a local hypoxic situation, presence of bacteria, minimal cell proliferation, and a reduced production of extracellular matrix. In our experience, by using the ozonated oil *in vivo*, the “restitutio ad integrum” including the final healing and scar tissue remodeling takes much less time in elderly and/or diabetic patients without any generalized or local side effects [13]. 

We have tested typical microorganisms as representative of either Gram-positive or Gram-negative aerobic bacteria often detected in human wounds and ulcers with slow tendency to heal. The experimental method that has been used was selected because other procedures (such as depositing the ozonated oil in small wells) did not reliably work due to the poor diffusion of ozonated oil throughout the medium. Reproducibility of results was excellent, and the experimental method closely reproduced the *in vivo* situation when the ozonated oil is applied (usually twice daily) on the ulcers.

One point that needs to be emphasized is that, before the oil application, the damaged skin surface must be cleaned by removing necrotic tissue, pus, loose fibrin deposition, and excess of fluid exudates. Such a cleaning operation can be done by curettage and washing the surface, preferably with ozonated water or diluted H_2_O_2_ solution, useful to eliminate most of the plasma proteins, hence antioxidants, which will limit the disinfection and the healing stimulation. How ozonated oil precisely acts remains a debatable question. According to the SEM results, the anti-infective activity is not dependent on structural alterations at the level of microorganisms. However, it seems likely that 1,2,4-trioxolane present in the ozonated oil, when added to the warm exudates film of the ulcer, slowly decomposes generating local oxygen, H_2_O_2_ as reactive oxygen species (ROS), and a trace of lipid oxidation products (4-HNE). Such a cascade can explain the prolonged disinfectant action and stimulation of proliferative activity of fibroblasts and keratinoblasts [12]. Other relevant questions are: (i) how much oil should be used? As the application is repeated every 12 hours, an oil layer of about 2 mm is enough; (ii) at what ozonation degree? The purpose of preparing a “weak”, “medium”, and “strong” oil reflects the need of treating either small, ample, or very infected ulcers. Thus, it is supposed that as an ulcer progressively improves, ozonated oil with lower grade of peroxide will be used. 

It is unfortunate that the topical use of cleaning the ulcer and the application of ozonated oil remain mostly confined to a few countries which have become knowledgeable of the ozone derivatives efficacy. Moreover, in most cases, the topical use takes place on the oil ozone derivative as such. Prospectively, it would be desirable to develop ointments characterized by both optimized skin permeability and safety upon open wounds. It is regrettable that the established medical community, which so far prefers to use antibiotic ointments in the absence or in the presence of growth factors or other methods [13], is not aware of the ozonated oil advantages as low-cost and great efficacy. As soon as it will be discovered, the topical treatment of torpid ulcers and wounds will be benefited by millions of patients, particularly in poor countries.

## Figures and Tables

**Table 1:** Chemical-physical characterization of the various samples (see text for further details).

Sample	PV (mEq/1,000 g)	AV (mg KOH/g)	IV (g/100 g)	Viscosity (mPa·s)	
				22°C	35°C
SO	198 ± 9	0.70 ± 0.01	113.65 ± 1.50	59.9 ± 1.1	34.2 ± 0.3
OSO low	949 ± 33	1.67 ± 0.08	96.05 ± 3.53	84.9 ± 0.7	48.1 ± 0.4
OSO middle	1631 ± 64	2.45 ± 0.05	81.32 ± 2.98	116 ± 1	64.5 ± 0.2
OSO high	3170 ± 101	7.32 ± 0.20	57.21 ± 2.34	248 ± 2	129 ± 2

PV: peroxide value; AV: acidity value; IV: iodine value.

**Table 2:** Viability (%) of the different strains as obtained with respect to control (microbial count in the presence of the corresponding amount of SO; see text for further details).

Type	Treatment time	25 mg OSO/5 mL of microorganism suspension			50 mg OSO/5 mL of microorganism suspension		
		l-OSO	m-OSO	h-OSO	l-OSO	m-OSO	h-OSO
*S. aureus *	1 h	65	58	58	57	47	47
	3 h	20	21	0.2	1	3.6	0.2
	6 h	0	0	0	0	0	0

*P. aeruginosa *	1 h	100	100	100	100	100	25
	3 h	100	100	5.7	13.7	15.1	2
	6 h	5	5	0	1.8	3.8	0

*E. faecalis *	1 h	100	100	100	100	100	100
	3 h	100	100	100	100	27	23
	6 h	20	15	0.1	7	0.1	0

*E. coli *	1 h	100	100	100	100	100	100
	3 h	0.8	0.8	0.5	0.4	0.4	0.4
	6 h	0.4	0.4	0	0.3	0.2	0

*C. albicans *	1 h	100	100	100	100	100	100
	3 h	39	38	18	13	13	0.2
	6 h	1.6	0	0	0	0	0
